# *Asparagusofficinalis* potentially supports cancer care: a systematic review of randomized and non-randomized clinical studies

**DOI:** 10.3389/fnut.2026.1621710

**Published:** 2026-03-12

**Authors:** Chen Shen, Xiao-Ti Wu, Xue-Feng Wang, Zhi-Jie Wang, Zi-Yu Tian, Nicola Robinson, Jian-Ping Liu

**Affiliations:** 1 Centre for Evidence-Based Chinese Medicine, Beijing University of Chinese Medicine, Beijing, China; 2 The Oncology Department, Shanxi Hospital of Traditional Chinese Medicine, Taiyuan, China; 3 Institute of Acupuncture and Moxibustion, China Academy of Chinese Medical Sciences, Beijing, China; 4 Institute of Health and Social Care, London South Bank University, London, United Kingdom; 5 Center for Evidence-Based Medicine, Hospital of Chengdu University of Traditional Chinese Medicine, Chengdu, Sichuan, China

**Keywords:** adjuvant therapy, Asparagaceae, bioactive compounds, cancer care, integrative oncology, quality of life, survival, vegetable

## Abstract

**Objective:**

To evaluate effectiveness and safety of *Asparagus officinalis* in cancer care.

**Methods:**

PubMed, the Cochrane Library, EMBASE, Web of Science, Scopus and four Chinese databases were searched up to January 9, 2025. Randomized and non-randomized clinical studies, cohort studies, or case-control studies were included for cancer patients using *A. officinalis* products alone or combined with conventional treatments. Primary outcomes were survival, response rates, and quality of life (QoL). GRADE approach was used to assess evidence certainty.

**Results:**

Ten studies (seven randomized trials, two non-randomized studies, one cohort study) with 8,898 participants were included. Compared to chemotherapy alone, *A. officinalis* granules plus chemotherapy improved survival-based effective rate [two studies, risk ratio (RR) 1.55, 95% confidence interval (CI) (1.24, 1.92), low certainty] and QoL-based effective rate [three studies, RR 1.76, 95% CI (1.47, 2.11), low certainty]. The objective response rate (ORR) was improved when chemotherapy was combined with either *A. officinalis* granules or syrup [four studies, RR 1.88, 95% CI (1.43, 2.48), low certainty]. *A. officinalis* products (syrup, granules or oral liquid) plus chemotherapy or radiotherapy, all found to have effects in improving immune function as CD4 and CD4/CD8. *A. officinalis* oral liquid combined with chemotherapy was associated with fewer adverse events, nausea and vomiting, and myelosuppression.

**Conclusion:**

*A. officinalis* products may improve survival, ORR, QoL and immune function as a complementary add-on therapy. Despite the limited number of studies and low certainty of evidence, the observed signals indicate a need for verification in well-designed, cancer-type–specific trials, particularly in lung cancer, using standardized, well-characterized extracts to establish definitive clinical applications and dosing protocols.

**Systematic review registration:**

The review protocol was registered with the International Prospective Register of Systematic Reviews (PROSPERO, CRD42025646003).

## 1 Introduction

Cancer is a formidable global health challenge that continues to pose a significant threat to human health. Cancer incidence has been rising steadily because of not only increasing life expectancy but also poor lifestyle choices. According to the latest data from the International Agency for Research on Cancer (IARC), an estimated 19.3 million new cancer cases and nearly 10 million cancer-related deaths occurred worldwide in 2020, implying that roughly 1 in 5 people will develop cancer during their lifetime (1). Recent forecasts further indicate that the global cancer burden will continue to rise rapidly, with the number of new cases projected to reach 35 million by 2050, largely driven by population growth and aging (2). Lifestyle-related risk factors such as tobacco use, unhealthy diet characterized by low fruit and vegetable intake, physical inactivity, and excess body weight account for a substantial proportion of cancer cases and deaths worldwide, underscoring the critical role of preventive strategies in reducing cancer burden (3–5). Additional synthesis of global data indicates that the rise in cancer will not be uniform and that countries with lower resources are likely to face larger proportional increases and more constrained service capacity (6, 7).

In parallel with the advancement of conventional cancer therapies, there has been increasing scientific and clinical interest in complementary and integrative approaches, including the use of natural products and traditional modalities, as potential sources of novel anti-cancer agents or as adjuncts to standard treatments. These strategies aim not only to enhance therapeutic efficacy and overcome drug resistance but also to mitigate treatment-related toxicities and improve patients’ QoL (8–10). Current guidance documents recommend selected integrative interventions such as mindfulness programs, tai chi or qigong, and acupuncture for defined indications in order to reduce fatigue and anxiety and to improve QoL, although availability remains inconsistent (11, 12). Natural product research continues to contribute to the development of anticancer agents (13).

Within the framework of dietary and plant-derived strategies, *A. officinalis*, a widely consumed vegetable, has attracted increasing attention research due to its rich composition of bioactive compounds. It contains a variety of phytochemicals, including flavonoids, saponins, and polysaccharides, which have been reported to possess multiple biological activities relevant to cancer prevention and management (14). In human diets, *A. officinalis* is commonly consumed raw in salads and after cooking such as steaming, boiling, sautéing and grilling (15, 16). It is used in soups and served alongside meat and fish (15). In addition, *A. officinalis* is incorporated into traditional cooked dishes such as fillings for boiled dumplings, as documented in a Chinese invention patent (17). Using a domestic-style boiling protocol (10 min; cooking water fully absorbed), Di Matteo et al. (16) showed increased extractability of key spear flavonols, particularly rutin, and that, after INFOGEST simulated gastrointestinal digestion, a fraction of the polyphenols remained bioaccessible (≈12.5–18.9%; 146.95–454.58 mg/kg dw).

Flavonoids, the major bioactive components of *A. officinalis*, have been investigated extensively for their anticancer properties. They can inhibit the growth and proliferation of cancer cells by modulating various signaling pathways involved in cell cycle regulation, apoptosis, and angiogenesis. For instance, Jiang et al. (18) demonstrated that quercetin could induce apoptosis in breast cancer cells by activating the caspase-dependent apoptotic pathway and inhibit the over-activated PI3K/Akt/mTOR signaling pathway. Independent studies showed that quercetin can remodel the tumor microenvironment by enhancing T and NK cell activity, reducing myeloid derived suppressor cell activity, and attenuating cancer glycolysis which supports a combined cytotoxic and immunomodulatory rationale for plant derived polyphenols (19). Rutin is the most abundant in *A. officinalis*, accounting for 60–80% of total phenolics. *In vitro* studies have demonstrated that rutin exerted significant inhibitory effects on the growth of colorectal cancer (HCT-116) and breast cancer (MCF-7) cells (20). Additionally, Paudel et al. (21) reported that rutin could suppress the migration and invasion of lung cancer cells by downregulating the expression of matrix metalloproteinases (MMPs), key enzymes involved in tumor metastasis. It has been well demonstrated that improving its bioavailability to enhance *in vivo* efficacy has become a key scientific issue for the clinical translation. For this, colon targeted compression coated tablets and pH sensitive nanospheres improved rutin solubility and site specific delivery and increased *in vitro* cytotoxicity which addresses a key barrier to translation (22, 23). In lung tumor models, liquid crystalline nanoparticles carrying rutin decreased proliferation and migration and increased apoptosis, which further supports importance of a delivery (24).

Saponins in *A. officinalis* also exhibit anti-cancer activities. They can promote apoptosis and cell cycle arrest. Additionally, saponins in *A. officinalis* interfere with cell cycle progression, cause mitochondrial dysfunction, increase Ca^2+^ concentration and ROS levels, and reduce mitochondrial membrane potential. Both endoplasmic reticulum (ER) dysfunction and DNA damage are triggered, which intensifies cellular stress, impairs protein homeostasis and genomic integrity, and escalates checkpoint and quality-control signaling. These regulatory mechanisms can eventually trigger caspase-dependent apoptosis, autophagy, and inhibition of cell migration (25). Polysaccharides of *A. officinalis* have been reported to possess immunomodulatory and anti-cancer effects. They can enhance the activity of macrophages, which play crucial roles in the innate immune response against cancer (17). Furthermore, polysaccharides of *A. officinalis* inhibits the migration, invasion and angiogenesis of hepatocellular carcinoma cells by targeting the HIF-1α/VEGF signaling pathway (26).

*Asparagus officinalis* by-products such as stems and roots are rich in dietary fiber and inulin, which exhibit prebiotic properties that improve gut microbiota composition and indirectly influence immune function and cancer prevention (15, 27). As indicated in clinical studies, lignans have shown a potential protective effect on cancer. Specifically, higher levels of dietary lignans were associated with a reduced risk of breast cancer, particularly among premenopausal women with at least one A2 allele of the CYP17 genotype (28).

However, translating these promising phytochemicals into clinically relevant applications has significant challenges. The anticancer effects observed in preclinical models fail to replicate in human trials due to low bioavailability, complex compound interactions within whole-plant extracts, and the methodological gap between cell-line/animal studies and human pathophysiology (29–31). Thus, it is highly desired to strengthen the literature through standardization of materials and preparations, clinically meaningful, patient-centered outcomes that are consistent with integrative oncology guidance.

A comprehensive systematic review of existing randomized and non-randomized clinical studies on the use of *A. officinalis* for cancer care is urgently needed, which will support understanding of the effectiveness and safety of *A. officinalis* in cancer management and gaps between its future clinical trials and therapeutic development.

## 2 Methods

### 2.1 Study design and registration

We conducted a systematic review and meta-analysis to assess the effects of *A. officinalis* on cancer care, synthesizing data from both randomized and non-randomized clinical studies. The review protocol was registered with the International Prospective Register of Systematic Reviews (PROSPERO, CRD42025646003) and adhered to the Preferred Reporting Items for Systematic Reviews and Meta-Analyses (PRISMA) guidelines (32).

### 2.2 Information sources and search strategy

We performed a comprehensive search of PubMed, the Cochrane Library, EMBASE, Web of Science, Scopus, China National Knowledge Infrastructure (CNKI), Wanfang database, Chinese Scientific Journal Database (VIP), and Sinomed from their inception to January 9, 2025. Additionally, we hand-searched the reference lists of all full-text papers for additional relevant reports. A supplementary search was also conducted in SciFinder to capture eligible literature. The search strategy is shown in Appendix 1.

### 2.3 Eligibility criteria

We included randomized controlled trials (RCTs), non-randomized controlled trials (non-RCTs), cohort studies, and case–control studies with no language restrictions. Participants included both: (a) adults already diagnosed with cancer or pre-cancerous lesions; and (b) undiagnosed individuals from studies investigating cancer incidence or prevention strategies. Interventions/exposures involved *A. officinalis* products, including *A. officinalis* alone, *A. officinalis* extracts, or products containing *A. officinalis*. These products have been used either alone or in combination with conventional medicine or other complementary therapies. The various forms of *A. officinalis* products may include syrup, granules, oral liquid, compound preparations, extracts, polysaccharides, and saponins. Comparisons included placebo, no intervention, conventional medicine, or other complementary therapies. The primary outcomes were cancer incidence and key clinical outcomes, including survival, response rates, and QoL. Secondary outcomes included symptom evaluation (e.g., pain relief), immune function (e.g., CD3, CD4, CD8), and safety outcomes (e.g., adverse events).

### 2.4 Study selection and data extraction

After removing duplicates, two authors (X-TW and X-FW) independently screened the titles and abstracts of the identified studies, followed by full-text screening for potentially eligible studies. Discrepancies were resolved through consensus, with arbitration by a third author (CS) if necessary. Data were extracted from the included studies by three authors (X-TW, X-FW, and CS) using a pre-designed extraction form. Extracted data included study characteristics (e.g., study design, sample size, participant demographics), intervention/exposure details, comparison details, outcomes and outcome measures.

### 2.5 Quality assessment

The risk of bias assessment was conducted using the Cochrane Risk of Bias 2.0 (RoB 2.0) tool (33) for RCTs, the Risk of Bias in Non-randomized Studies of Interventions (ROBINS-I) tool (34) for non-randomized trials, and the Newcastle-Ottawa Scale (NOS) (35) for cohort and case-control studies. For RCTs, bias was evaluated across five domains: the randomization process, deviations from intended interventions, missing data, measurement of outcomes, and selection of the reported result. For non-RCTs, bias was assessed across seven domains: confounding, selection of participants, classification of interventions, deviations from intended interventions, missing data, measurement of outcomes, and selection of the reported result. The NOS, a widely used tool for assessing the quality of observational studies, evaluates studies based on three domains: selection, comparability, and outcome. Two authors independently assessed the risk of bias for each study, with discrepancies resolved through consensus and arbitration by a third author if necessary.

We utilized the Grading of Recommendations Assessment, Development, and Evaluation (GRADE) approach to evaluate the certainty of evidence for the primary outcomes in meta-analyses (36). The GRADE approach assesses the certainty of evidence as high, moderate, low, and very low. Key dimensions of the assessment included risk of bias, which evaluates potential systematic errors in study design and conduct; imprecision, which considers the precision of effect estimates and factors like sample size and CIs; inconsistency, which examines variability in results across studies; indirectness, which assesses the applicability of evidence to the population, intervention, or outcome of interest; and publication bias, which looks at the potential for selective reporting or non-publication of studies.

### 2.6 Data synthesis and analysis

The data synthesis and analysis were conducted using a combination of meta-analysis and narrative synthesis. When two or more studies were sufficiently homogeneous, a meta-analysis was performed to pool the data and generate summary estimates. This involved calculating RRs with 95% CIs for dichotomous outcomes and mean differences (MDs) with 95% CIs for continuous outcomes. Heterogeneity was assessed using the *I*^2^ test and *χ*^2^ test, and a random-effects model was used to account for potential heterogeneity between studies. The *I*^2^ value was interpreted using the rough guide proposed by the Cochrane Handbook: 0 to 40% might not be important; 30 to 60% may represent moderate heterogeneity; 50 to 90% may represent substantial heterogeneity; and 75 to 100% may represent considerable heterogeneity. Subgroup analyses were conducted to explore the impact of potential effect modifiers on the effects of the intervention, such as different types of cancer or pre-cancerous lesions, different interventions, different study designs and time points. Publication bias was assessed using funnel plots and Egger’s test when there were a sufficient number of studies (generally considered to be at least 10 studies) included in the meta-analysis. When the studies were too heterogeneous, a narrative synthesis was conducted to describe and compare the findings across studies.

## 3 Results

### 3.1 Description of the included studies

**Figure 1:**
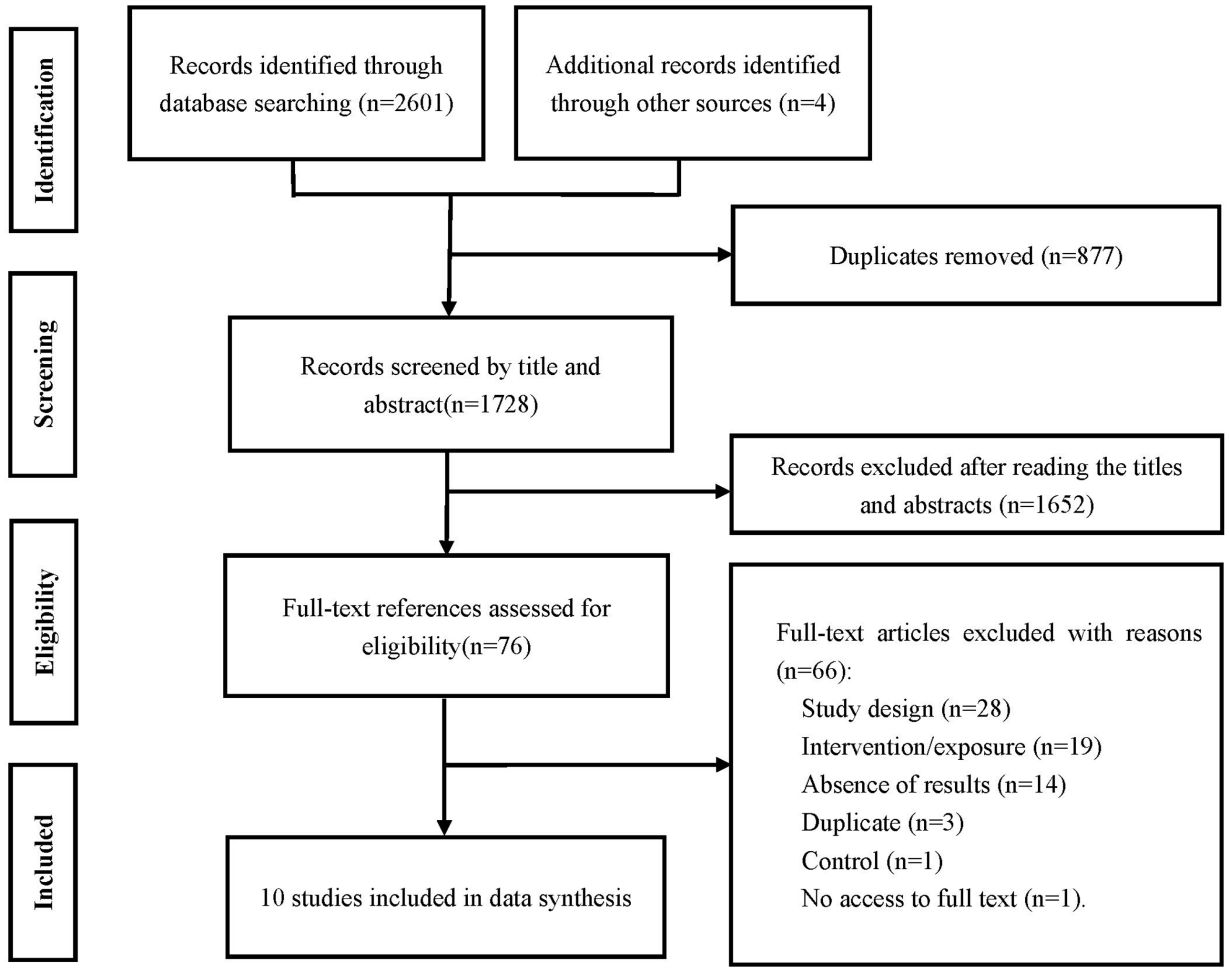
The PRISMA flow of literature search and selection of studies.

A total of 2,605 records were identified, and finally, 10 studies were included in the data synthesis (Figure 1) (37–46). Table 1 provides an overview of the included studies. The studies included seven randomized trials (39, 41–46), two non-randomized studies (37, 40), one cohort study (38). A total of 8,898 participants were involved and the sample sizes ranged from 36 (37) to 7,995 (38). Nine studies involved lung cancer participants (37–44, 46). Specifically, six articles investigated various types of lung cancer (38, 39, 41, 43, 44, 46), while the other three studies (37, 40, 42), published in the 1990s, included multiple types of cancer. Interventions/exposures used in the included studies mainly consisted of *A. officinalis* granules (39, 41, 44, 46), *A. officinalis* oral liquid (43), and *A. officinalis* syrup (37, 40, 42, 45), all prepared from fresh *A. officinalis* as the main ingredient. In addition, other ingredients may be included depending on the specific product formulation. For example, *A. officinalis* oral liquid may also contain honey and licorice, while *A. officinalis* syrup may contain sugar and water. The duration of intervention/exposure varied from 1 to 2 months.

**Table 1:** Characteristics of the included studies.

ID	Study type	Age (mean ± SD)		Sample size	Types of cancer or precancerous lesions	Intervention/exposure	Composition of *A. officinalis* products	Control	Duration of I/E (months)
		I/E	C						
Li XZ 1994 (37)	Non-RCT	NR	NR	36	Lung cancer, esophageal cancer, gastric cancer, and breast cancer	*A. officinalis* syrup + chemotherapy	*A. officinalis* juice 50–75, sugar 15–25, water 8–25, additives 0.5–5	Chemotherapy	NR
Lv QJ 2000 (38)	Cohort study	≥40	≥40	7995	Lung cancer	*A. officinalis* vegetables	*A. officinalis* vegetables	NR	NR
Pang YL 2016 (39)	RCT	48.6 ± 2.5	49.5 ± 1.8	120	Lung cancer	*A. officinalis* granules + chemotherapy	Extract of fresh *A. officinalis*	Chemotherapy	2
Wang BC 1996 (40)	Non-RCT	20~71 (mean: 49.6)		166	Lung cancer (*n* = 65), esophageal cancer (*n* = 40), gastric cancer (*n* = 15), colorectal cancer (*n* = 8), lymphoma (*n* = 9), breast cancer (*n* = 11), nasopharyngeal cancer (*n* = 3), and others (*n* = 15)	*A. officinalis* syrup + radiotherapy or chemotherapy	*A. officinalis* juice 50–75, sugar 15–25, water 8–25, additives 0.5–5	Fuzheng granules + radiotherapy or chemotherapy	1
Wang FW 2005 (41)	RCT	46~75 (mean: 59.36)	45~70 (mean: 57.2)	84	Lung cancer [squamous cell carcinoma (*n* = 42), adenocarcinoma (*n* = 31), small cell carcinoma (*n* = 8), and large cell carcinoma (*n* = 3)]	*A. officinalis* granules + chemotherapy	Extract of fresh *A. officinalis*	Chemotherapy regimen	1.5
Wang JY 1996 (42)	RCT	58.6		50	Lung cancer (*n* = 28), gastric cancer (*n* = 15), breast cancer (*n* = 5), nephroblastoma (*n* = 1), and esophagus cancer (*n* = 1)	*A. officinalis* syrup + chemotherapy		*A. officinalis* juice 50–75, sugar 15–25, water 8–25, additives 0.5–5	>1
Xie XY 2017 (43)	RCT	52–76	54–72	57	Non-small cell lung cancer [squamous cell carcinoma (*n* = 30), adenocarcinoma (*n* = 27)]	*A. officinalis* oral liquid + chemotherapy	Fresh *A. officinalis*	Chemotherapy	1.5
Xu S 2008 (44)	RCT	Chemotherapy group: 40~66 (mean: 56)	Chemotherapy group: 40~65 (mean: 55)	85	Non-small cell lung cancer	Chemotherapy group: *A. officinalis* granules + chemotherapy (*n* = 22)	Stock solution of *A. officinalis*	Chemotherapy group: chemotherapy (*n* = 23)	NR
		Radiotherapy group: 45~72 (mean: 52)	Radiotherapy group: 46~70 (mean: 53)			Radiotherapy group: *A. officinalis* granules + radiotherapy (*n* = 20)		Radiotherapy group: radiotherapy (*n* = 20)	
Yu RL 1996 (45)	RCT	41–76	41–76	128	Esophageal cancer	Concentrated *A. officinalis* syrup + radiotherapy	*A. officinalis* juice 50–75, sugar 15–25, water 8–25, additives 0.5–5	Radiotherapy	12
Zhuang HX 2008 (46)	RCT	35~74 (58.6)	38~70 (56.2)	168	Primary lung cancer [squamous cell carcinoma (*n* = 83), adenocarcinoma (*n* = 54), small cell carcinoma (*n* = 16), and large cell carcinoma (*n* = 5)]	*A. officinalis* granules + chemotherapy	Extract of fresh *A. officinalis*	Chemotherapy regimen	2

RCT, randomized controlled trial; I/E, Interventions/exposures; *A. officinalis*: *Asparagus officinalis*.

### 3.2 Risk of bias evaluation

**Figure 2:**
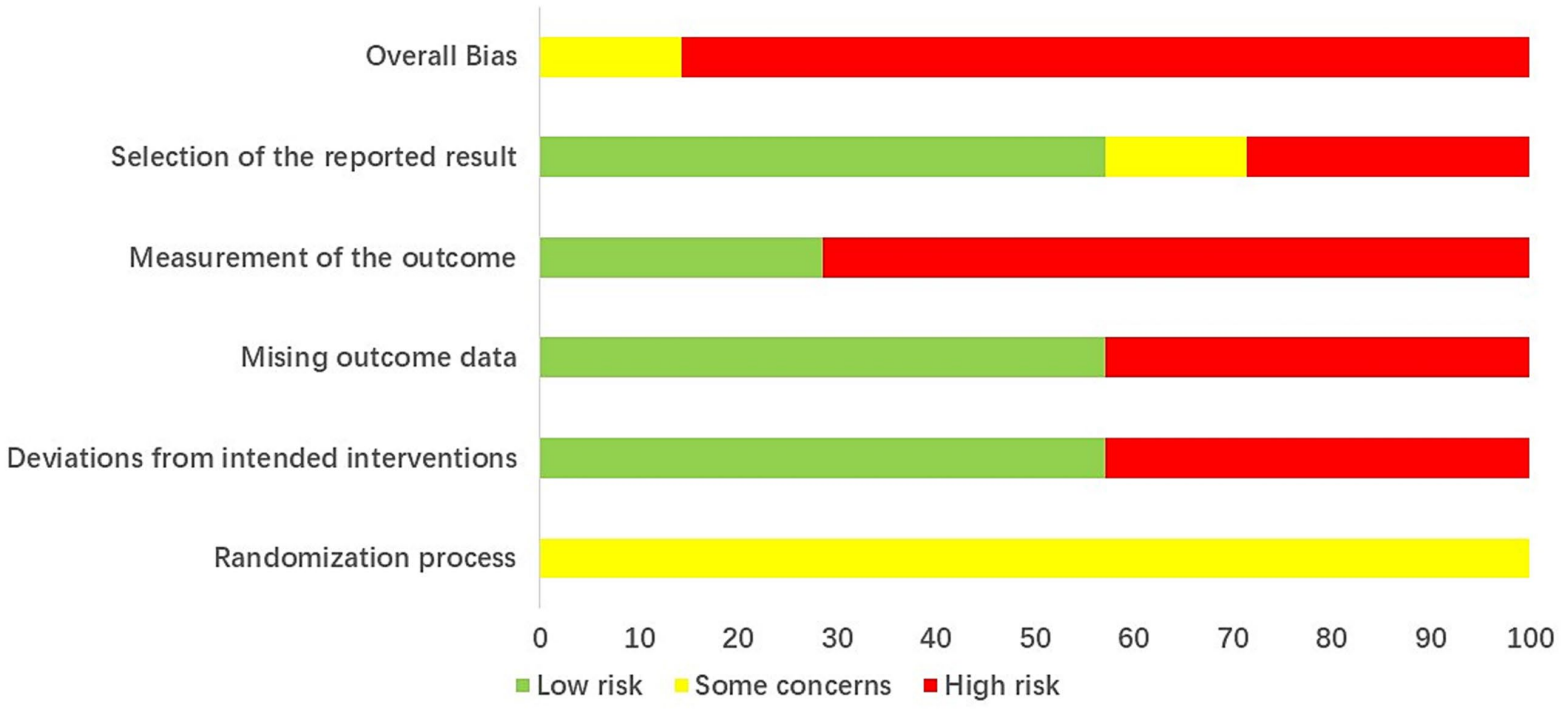
Risk of bias assessment result of RCTs.

The risk of bias assessment of RCTs are shown in Figure 2. In the seven included RCTs, three did not mention the specific randomization method (42, 44, 45), and all seven did not provide information on randomization concealment (39, 41–46). Additionally, three studies did not address whether the baselines were comparable (42, 44, 45). These issues suggest that there are “some concerns” regarding the randomization process. None of the studies used a placebo control, and blinding was not applied. Only one study reported drop-outs and loss-to-follow-up (43). In two studies, the number of participants reported in the methods and results sections was inconsistent (43, 44). One study had missing information that prevented the determination of the planned sample size in the protocol (45). As a result, these three studies were assessed as having bias risks in deviations from intended interventions, missing outcome data, and selection of the reported result. Moreover, five studies employed patient-reported outcomes, which leads to a high risk of bias in measurement of the outcome (39, 41, 42, 45, 46). The results of methodological quality evaluation of non-RCT and cohort study are presented in Supplementary File 2.

### 3.3 Primary outcomes

#### 3.3.1 Cancer incidence

A cohort study (38) on Yunnan Tin Corporation miners reported cancer incidence and explored the link between vegetable intake and lung cancer risk. Using a Food Frequency Questionnaire and 24 h food recall for 27 vegetables, it found that The reproducibility of *A. officinalis* intake frequency is good. Some vegetables, such as wax gourd and celery, showed a negative correlation with lung cancer risk. However, whether *A. officinalis* serves as a protective factor remains unreported. Without specific data, its role in reducing lung cancer risk remains undetermined.

#### 3.3.2 Survival

##### 3.3.2.1 One-year overall survival (OS) rate

In the meta-analysis of one RCT (45), low certainty of evidence (Supplementary Table S3, outcome measure “One-Year Survival Rate”) showed that the combination of *A. officinalis* syrup and radiotherapy significantly improved the one-year overall survival rate compared to radiotherapy alone (Table 2). The RR for the one-year survival rate was 1.62 with a 95% CI of [1.14, 2.28].

**Table 2:** Meta-analysis results of primary outcomes.

Outcome and outcome measure	Effect size	95% CI
Outcome: survival		
Outcome measure: one-year survival rate		
Comparison: *A. officinalis* syrup + radiotherapy vs. radiotherapy (*N* = 128, one RCT)	RR 1.62	1.14 to 2.28
Outcome measure: survival-based effective rate		
Comparison: *A. officinalis* granules + chemotherapy vs. chemotherapy (*N* = 252, two RCTs)	RR 1.55	1.24 to 1.92
Outcome: response rate		
Outcome measure: ORR	RR 1.88	1.43 to 2.48
Comparison: *A. officinalis* granules + chemotherapy vs. chemotherapy (*N* = 372, three RCTs)	RR 2.00	1.46 to 2.74
Comparison: *A. officinalis* syrup + chemotherapy vs. chemotherapy (*N* = 50, one RCT)	RR 1.57	0.91 to 2.72
Outcome measure: DCR		
Comparison: *A. officinalis* syrup + chemotherapy vs. chemotherapy (*N* = 50, one RCT)	RR 1.15	0.95 to 1.40
Outcome: QoL		
Outcome measure: KPS		
Comparison: *A. officinalis* syrup + chemotherapy vs. chemotherapy (*N* = 57, one RCT)	MD 7.95	3.83 to 12.07
Outcome measure: quality-of-life-based effective rate		
Comparison: *A. officinalis* granules + chemotherapy vs. chemotherapy (*N* = 372, three RCTs)	RR 1.76	1.47 to 2.11

RCT, randomized controlled trial; *A. officinalis*, *Asparagus officinalis*. CI, confidence interval; MD, mean difference; RR, risk ratio; ORR, objective response rate; DCR, disease control rate; QoL, quality of life; KPS, Karnofsky performance scale.

##### 3.3.2.2 Survival-based effective rate

The two of RCTs (41, 46) applied specific criteria to evaluate the survival-based effective rate. These criteria defined effective response based on the duration of survival after intervention, with different thresholds for patients at different disease stages. For instance, Stage II patients were considered to have effective response if they lived more than 6 months after intervention, while those with venous tumor thrombus or with single/multiple tumors with a total diameter of ≥10 cm needed to survive more than 3 months. Stage III patients required survival of more than 2 months to be classified as effective.

The low certainty of evidence (Supplementary Table S3, outcome measure “Survival-Based Effective Rate”) demonstrated that the addition of *A. officinalis* granules to chemotherapy significantly enhanced the survival-based effective rate, in lung cancer participants, compared to chemotherapy alone (Table 2). The RR for the survival-based effective rate was 1.55 with a 95% CI of [1.24, 1.92].

#### 3.3.3 Response rate

##### 3.3.3.1 Objective response rate (ORR)

The low certainty of evidence (Supplementary Table S3, outcome measure “ORR”) on ORR showed a significant improvement in patients receiving *A. officinalis* products in combination with chemotherapy compared to chemotherapy alone [Table 2, four studies (39, 41, 42, 46), RR 1.88, 95% CI (1.43, 2.48)]. The pooled RR for *A. officinalis* granules was 2.00 [372 lung cancer participants, 95% CI (1.46–2.74)], while for *A. officinalis* syrup, the RR was 1.57 [95% CI (0.91–2.72), *p* = 0.11].

##### 3.3.3.2 Disease control rate (DCR)

One study (42) was included in the evaluation of the DCR. The study compared the combination of *A. officinalis* syrup and chemotherapy with chemotherapy alone. The very low certainty of evidence (Supplementary Table S3, outcome measure “DCR”) showed a RR of 1.15 with a 95% CI of [0.95, 1.40] (Table 2).

##### 3.3.3.3 Median response rate

In the non-RCT (37) comparing the combination of *A. officinalis* and chemotherapy with chemotherapy alone for patients with advanced tumors such as lung cancer, esophageal cancer, and gastric cancer, the median response rate of the *A. officinalis*-plus-chemotherapy group was 39%, while that of the chemotherapy-only group was 30.6%. The non-RCT observed a significant statistical difference between the two groups; however, the RR and CI could not be calculated due to the lack of reported sample size in the study.

#### 3.3.4 Quality of life

##### 3.3.4.1 Karnofsky performance scale (KPS)

Table 2 presents the results of a study by Xie (43) comparing the KPS between patients receiving *A. officinalis* syrup combined with chemotherapy and those receiving chemotherapy alone. The MD in Karnofsky Score was 7.95 with a 95% CI of [3.83, 12.07]. The certainty of evidence was low (Supplementary Table S3, outcome measure “Karnofsky Score”).

##### 3.3.4.2 Quality-of-life-based effective rate

The QoL-based effective rate was defined in three RCTs (39, 41, 46) with an increase of 10 points of Karnofsky Score for effectiveness, and no improvement or a decrease for ineffectiveness. The three RCTs evaluated the QoL-based effective rate between lung cancer patients receiving *A. officinalis* granules combined with chemotherapy and those receiving chemotherapy alone (Table 2). The RR for the QoL-based effective rate was 1.76 with a 95% CI of [1.47, 2.11] and the certainty of evidence was low (Supplementary Table S3, outcome measure “quality-of-life-based effective rate”).

### 3.4 Secondary outcomes

#### 3.4.1 Symptom evaluation

One non-RCT (40) focused on evaluating the remission rate of subjective symptoms in cancer patients undergoing radiotherapy or chemotherapy. The remission rate of subjective symptoms was calculated based on the proportion of patients in each group whose symptoms of nausea, vomiting, abdominal distension, and loss of appetite caused by radiotherapy or chemotherapy improved. The meta-analysis suggested that *A. officinalis* syrup was comparable to Fuzheng granules in alleviating these subjective symptoms during radiotherapy or chemotherapy for cancer patients [Table 3, RR 1.03, 95% CI (0.91, 1.16)].

**Table 3:** Meta-analysis results of symptom evaluation and safety outcome.

Outcome and outcome measure	Effect size	95% CI
Outcome: symptom evaluation		
Outcome measure: remission rate of subjective symptoms		
Comparison: *A. officinalis* syrup + radiotherapy or chemotherapy vs. Fuzheng granules + radiotherapy or chemotherapy (*N* = 166, 1 non-RCT (40))	RR 1.03	0.91 to 1.16
Outcome: safety outcome		
Outcome measure: incidence of nausea and vomiting		
Comparison: *A. officinalis* oral liquid + chemotherapy vs. chemotherapy – 3 weeks (*N* = 55, one RCT (43))	RR 0.65	0.44 to 0.96
Comparison: *A. officinalis* oral liquid + chemotherapy vs. chemotherapy – 6 weeks (*N* = 57, one RCT (43))	RR 0.58	0.39 to 0.86
Outcome measure: incidence of myelosuppression		
Comparison: *A. officinalis* oral liquid + chemotherapy vs. chemotherapy—3 weeks (*N* = 57, one RCT (43))	RR 0.62	0.38 to 1.02
Comparison: *A. officinalis* oral liquid + chemotherapy vs. chemotherapy—6 weeks (*N* = 57, one RCT (43))	RR 0.54	0.33 to 0.91

RCT, randomized controlled trial; *A. officinalis*, *Asparagus officinalis*.

#### 3.4.2 Immune function

Meta analysis results are shown in Supplementary Table S4, meta-analysis results of immune function.

##### 3.4.2.1 CD3

In the radiotherapy setting, compared with radiotherapy alone, the CD3 levels were significantly higher in the group with *A. officinalis* granules combined with radiotherapy at 30 days [MD = 7.99, 95% CI (4.11, 11.87), 1 study (44)] and 45 days [MD = 12.9, 95% CI (9.82, 15.98), one study (44)]. In the chemotherapy scenario, the CD3 level was significantly higher in the group with *A. officinalis* granules combined with chemotherapy than in the chemotherapy-alone group at 60 days [MD = 17.26, 95% CI (15.42, 19.1), 2 studies (41, 46)].

##### 3.4.2.2 CD4

During radiotherapy, the CD4 levels were significantly higher in the group with *A. officinalis* granules combined with radiotherapy than in the radiotherapy-alone group at 30 days [MD = 7.41, 95% CI (3.73, 11.09), 1 study (44)] and 45 days [MD = 9.77, 95% CI (6.81, 12.73), 1 study (44)]. In chemotherapy, the CD4 levels were significantly higher in the groups with *A. officinalis* oral liquid combined with chemotherapy at 45 days [MD = 6.09, 95% CI (4.67, 7.51), 1 study (43)] and *A. officinalis* granules combined with chemotherapy at 60 days [MD = 8.6, 95% CI (6.42, 10.78), 2 study (41, 46)] compared to the chemotherapy-alone group.

##### 3.4.2.3 CD8 and CD4/CD8

With 45 days of chemotherapy, the CD8 level was significantly lower in the group with *A. officinalis* oral liquid combined with chemotherapy than in the chemotherapy-alone group [MD = 5.12, 95% CI (−6.43, −3.81), 1 study (43)].

In radiotherapy, the CD4/CD8 ratio was significantly higher in the group taking *A. officinalis* granules combined with radiotherapy than in the radiotherapy-alone group at 30 days (44) [MD = 0.38, 95% CI (0.03, 0.73)] and 45 days (44) [MD = 0.59, 95% CI (0.19, 0.99)]. For those receiving chemotherapy, the CD4/CD8 ratio was significantly higher in the groups taking *A. officinalis* oral liquid combined with chemotherapy at 45 days (43) [MD = 0.39, 95% CI (0.27, 0.51)] and *A. officinalis* granules combined with chemotherapy at 60 days (41, 46) [MD = 0.2, 95% CI (0.08, 0.33)] compared to the chemotherapy-alone group.

#### 3.4.3 Safety outcomes

This meta-analysis (Table 3) explored the safety of *A. officinalis* oral liquid combined with chemotherapy compared to chemotherapy alone. For nausea and vomiting, data from Xie (43) showed that at 3 weeks, the combined group had a lower risk [RR = 0.65, 95% CI (0.44, 0.96), *p* = 0.03], and at 6 weeks, the risk was also lower [RR = 0.58, 95% CI (0.39, 0.86), *p* = 0.007].

Regarding myelosuppression, over 3 weeks, there was a non-significant trend towards lower risk in the combined group [RR = 0.62, 95% CI (0.38, 1.02), *p* = 0.06], while over 6 weeks, the combined group had a significantly lower risk [RR = 0.54, 95% CI (0.33, 0.91), *p* = 0.02] (43).

## 4 Discussion

### 4.1 Summary of findings

This systematic review evaluated the effectiveness and safety of *A. officinalis* used in cancer care, synthesizing evidence from 10 clinical studies (37–46), including seven RCTs (39, 41–46), two non-RCTs (36, 40), and one cohort study (38). The findings suggested that compared to chemotherapy alone, *A. officinalis* granules plus chemotherapy significantly improved the survival-based effective rate [two RCTs (41, 46), RR 1.55, 95% CI (1.24, 1.92), low certainty of evidence] and QoL-based effective rate [three RCTs (37, 41, 46), RR 1.76, 95% CI (1.47, 2.11), low certainty of evidence], while *A. officinalis* products (*A. officinalis* granules or *A. officinalis* syrup) combined with chemotherapy improved ORR [four RCTs (39, 41, 42, 46), RR 1.88, 95% CI (1.43, 2.48), low certainty of evidence]. *A. officinalis* syrup plus chemotherapy or radiotherapy, *A. officinalis* granules plus chemotherapy or radiotherapy, *A. officinalis* oral liquid combined with chemotherapy were all found to have effects in improving immune function in cancer patients as CD4 and CD4/CD8. Additionally, *A. officinalis* oral liquid combined with chemotherapy were found associated with fewer adverse events like nausea and vomiting, and myelosuppression.

### 4.2 Agreements and disagreements with previous studies

The conclusions of this systematic review align with fundamental findings from preclinical research, and provide more direct evidence at the translational medicine level. At the mechanistic level, the immune-enhancing effects observed in this study, such as the improvement in CD4/CD8 ratios, are consistent with multiple studies reporting that *A. officinalis* polysaccharides and other components can activate macrophages and modulate cytokine activity (47). This immune-enhancing effect has been further corroborated at the mechanistic level. Studies have shown that *A. officinalis* polysaccharides activate macrophages through canonical innate immune pathways, including NLRP3 inflammasome signaling, and lead to increased nitric oxide and cytokine release *in vitro* as well as recovery of immune indices *in vivo* (47–49). On the other hand, the potential tumor-suppressive effects suggested by our findings also resonate with the observations of Shao et al. (50), who demonstrated that crude saponins from *A. officinalis* exhibit direct anti-proliferative activity against human leukemia HL-60 cells by irreversibly inhibiting DNA synthesis.

The mechanistic insights have emerged from studies focusing on specific bioactive components of *A. officinalis*. As noted in the introduction of this review, flavonoids, saponins, and polysaccharides extracted from *A. officinalis* can regulate multiple cancer-related pathways, including PI3K/Akt/mTOR signaling, MMP-mediated metastasis, mitochondrial apoptosis cascades, and HIF-1α/VEGF-driven angiogenesis (18, 20, 21, 25). Complementing these pathways, standardized *A. officinalis* stem extracts have been shown to dampen macrophage production of IL-6 and IL-1β through suppression of p44/42 MAPK and Akt phosphorylation, which provides a plausible immunologic basis for improved symptom control and treatment tolerance in supportive oncology settings (51–53). The findings of our study indicate that the combination of *A. officinalis* products with chemotherapy or radiotherapy may improve ORRs and survival outcomes, which may reflect the positive clinical manifestations of these multi-target mechanisms.

However, significant challenges remain in translating these promising preclinical findings into definitive clinical benefits, of which one involves L-asparaginase derived from *A. officinalis*. As retrospectively illustrated by Seeman (54), the selective cytotoxicity of asparaginase against cancer cells deficient in asparagine synthetase established its role as a cornerstone therapy for acute lymphoblastic leukemia. This provides a strong theoretical basis for *A. officinalis* as a potential natural source of asparaginase. Nevertheless, research by Mitra et al. (55) revealed complexities in this translation: shatavarins (containing shatavarin IV) isolated from the roots of *Asparagus racemosus* (a congeneric species related to *A. officinalis*) demonstrated significant anticancer activity both *in vitro* and *in vivo*, yet their mechanism appeared to emphasize direct cytotoxicity and immunomodulation rather than the asparaginase pathway. Taken together, current evidence suggests that the anti-cancer profile of *A. officinalis* is likely polypharmacologic, with immune modulation acting alongside direct anti-proliferative and anti-angiogenic effects rather than a single dominant pathway (56, 57).

However, our review has failed to find substantial benefits in *A. officinalis* syrup plus chemotherapy on ORR [one study, RR 1.57, 95% CI (0.91–2.72)] or on DCR [one study, RR 1.15, 95% CI (0.95, 1.40)]. As noted by Godsey and Grundmann (58), evidence supporting the use of *A. officinalis* as a complementary approach in oral cancer remains preliminary, and current findings are insufficient to justify clinical application without further well-designed studies. In addition, prebiotic fractions such as inulin-rich fructans from *A. officinalis* may contribute to immunoregulatory benefit through microbiome-derived short-chain fatty acids, which offers a testable axis for future patient-reported and inflammatory endpoints (59–61). These mechanistic possibilities align well with the overall conclusion of our systematic review, which collectively underscores the need for greater methodological standardization and precision in future research, as well as increased attention to patient-reported outcomes.

### 4.3 Strengths and limitations

This systematic review offers a comprehensive assessment of the role of *A. officinalis* in cancer care. By incorporating both randomized and non-randomized studies, it provides a more extensive view of the potential benefits of *A. officinalis* products. This comprehensive approach helps to capture a wider range of evidence, including different study perspectives that might be overlooked if only one type of study was considered. Moreover, the application of meta-analysis for key outcomes such as survival-based effective rate and QoL-based effective rate is a significant strength. Meta-analysis allows for the quantitative synthesis of data from multiple studies, which can increase the statistical power and precision of the overall estimates. This quantitative approach helps to draw more reliable conclusions about the effectiveness of *A. officinalis* in improving these outcomes, compared to relying on individual studies alone. Another strength is that the clinical effects observed in our synthesis corroborate the biological rationale emerging from mechanistic studies (56, 57, 62, 63), thereby providing further support for a stronger translational focus in future *A. officinalis* research.

However, several limitations should be acknowledged. One of the major drawbacks is the limited number of included studies and relatively small sample sizes. The small number of studies for certain outcomes, like the DCR, restricts the ability to draw firm conclusions. Small sample sizes yield unstable and less precise effect estimates and increase the likelihood that observed results reflect random variation rather than true effects (64). This can lead to uncertainty about the true effectiveness of *A. officinalis* in these areas. The methodological quality of some studies was suboptimal, with issues such as inadequate blinding, incomplete of reporting of outcomes and inconsistency of sample size reported throughout the article. These limitations may have introduced bias and reduced the reliability of the findings. Although our search was current, the publication dates of the included studies (1994–2017) are relatively old, which may affect the applicability of some effectiveness findings to current ultra-modern treatment contexts. Nevertheless, the potential supportive benefits of *A. officinalis* for QoL and immune function, rooted in its long history of dietary and traditional use, may remain relevant as a complementary approach to contemporary cancer care. It is also important to recognize formulation heterogeneity, as studies have used syrups, granules, oral liquids and standardized stem extracts with emerging evidence suggesting that different matrices may differentially influence cytokine signaling and macrophage responses (49, 51).

Another limitation is the significant clinical and methodological heterogeneity among the included studies. The studies varied widely in terms of the patient populations, study design, intervention types, and outcome measures. Notably, while most studies focused on lung cancer, there was heterogeneity in cancer types: three older studies (37, 40, 42) enrolled patients with various solid tumors, and even among lung cancer studies, specific subtypes and stages were often not detailed. This variability limits the generalizability of our findings to any specific cancer type or stage. Furthermore, the interventions exhibited considerable diversity. Different studies used various forms of *A. officinalis* products (e.g., granules, syrups, and oral liquids), which not only differed in dosages and durations of use but also likely in their precise formulation and composition of bioactive compounds, as excipients like honey, licorice, and sugar varied across products. Evidence have further suggested that such matrix differences may influence cytokine signaling and macrophage responses (49, 51). The combination of these factors, diverse cancer populations and non-standardized interventions, makes it challenging to isolate the true effect of *A. officinalis* and draws attention to the need for more homogeneous future research. Future studies would benefit from adopting standardized chemical characterization of *A. officinalis* products, including quantified flavonoids, saponins and polysaccharides, because such consistency would improve dose-response assessment and allow more meaningful comparisons across trials. Recent preclinical and food-science studies have begun to provide these detailed compositional data, offering a foundation for more homogeneous research in the future (65–67).

### 4.4 Implications for future research

The findings of this study highlight the potential of *A. officinalis* as a complementary therapy in cancer care, but also underscore the critical need for more rigorous research. To address the current limitations, future studies should prioritize large-scale, multi-center RCTs with placebo controls and adequate blinding, specifically targeting homogeneous patient populations defined by cancer type and stage. RCTs should adhere to CONSORT guideline and CONSORT for Chinese Herbal Medicine Formulas 2017 extension to ensure transparent and complete reporting (68, 69). Beyond studying purified extracts, it is feasible to develop *A. officinalis* into palatable, standardized functional foods, as evidenced by patents proposing food formulations that use asparagus as a primary functional component (17), and formulations that include asparagus within multi-herb combinations also offer ideas for dosage-form development (70). Incorporating translational components into trial design, such as collecting biospecimens for pharmacokinetic and pharmacodynamic analyses, would simultaneously elucidate mechanisms of action in human subjects while evaluating clinical efficacy. Furthermore, studies should implement extended follow-up periods to assess long-term safety and systematically document potential interactions with conventional cancer treatments. Through these methodologically robust approaches, future research can generate the high-quality evidence needed to definitively establish the role of *A. officinalis* in oncology practice.

## 5 Conclusion

In conclusion, this systematic review provides preliminary evidence supporting the potential benefits of *A. officinalis* in improving survival-based effective rate, QoL-based effective rate and enhancing immune function when used as a complementary therapy. However, several limitations temper these findings. The included studies had significant heterogeneity in design, intervention types, and outcome measures. Many studies had methodological flaws, including unclear randomization procedures and a high risk of bias for patient-reported outcomes. The limited number of studies and small sample sizes for some outcomes also restricted the reliability of the conclusions. For current clinical practice, our results suggest that *A. officinalis* could be considered as a supportive adjunct to conventional treatment, particularly for potential QoL and immune function benefits, rather than as a direct antitumor intervention. Patients interested in using these products should be advised to select standardized formulations and use them under medical supervision while continuing conventional therapies. Future rigorously designed trials focusing on specific cancer types and standardized extracts are needed to establish definitive clinical applications and dosing protocols.

## Data Availability

The original contributions presented in the study are included in the article/Supplementary material, further inquiries can be directed to the corresponding author.
