# Supplementary material for: Asparagusofficinalis potentially supports cancer care: a systematic review of randomized and non-randomized clinical studies
Source: Front Nutr. 2026 Mar 12;13:1621710. doi: 10.3389/fnut.2026.1621710 (PMC13017270; doi:10.3389/fnut.2026.1621710)
Supplement: Supplementary file 1 [file Table_1.DOCX]

**Appendix 1 Search strategy in PubMed**

#1 ("Neoplasms"[MeSH Terms]) OR ("cancer*"[Tiab]) OR ("neoplas*"[Tiab]) OR ("tumor*"[Tiab]) OR ("tumour*"[Tiab]) OR ("carcinoma*"[Tiab]) OR ("hodgkin*"[Tiab]) OR ("nonhodgkin*"[Tiab]) OR ("adenocarcinoma*"[Tiab]) OR ("leukemi*"[Tiab]) OR ("leukaemi*"[Tiab]) OR ("metasta*"[Tiab]) OR ("oncolog*"[Tiab]) OR ("lymphoma*"[Tiab]) OR ("malignan*"[Tiab]) OR ("sarcoma*"[Tiab]) OR ("myeloma*"[Tiab]) OR ("Abdominal Lumps"[Tiab])

#2 (“Asparagus”[Tiab]) OR ("Asparagus Plant"[Mesh]) OR (“Asparagus Plants”[Tiab]) OR (“Plant, Asparagus”[Tiab]) OR (“Plants, Asparagus”[Tiab]) OR (“Asparagus officinalis”[Tiab]) OR (“asparagus syrup”[Tiab]) OR (“Lusun TangJiang”[Tiab]) OR (“asparagus granules”[Tiab]) OR (“Lusun Keli”[Tiab]) OR (“asparagus oral liquid”[Tiab]) OR (“asparagus oral solution”[Tiab]) OR (“Lusun Koufuye”[Tiab]) OR (“asparagus saponins”[Tiab]) OR (“asparanin A”[Tiab]) OR (“asparagus polysaccharide”[Tiab]) OR (“Asparagosin”[Tiab]) OR (“asparagus officinalis polysaccharide”[Tiab]) OR (“asparagus flavones”[Tiab]) OR (“Compound asparagus preparation”[Tiab]) OR (“Fufang Lusun zhiji”[Tiab]) OR (“Asparagus mixture”[Tiab]) OR (“Fufang Lusun Heji”[Tiab]) OR (“Compound asparagus extract”[Tiab]) OR (“Asparagus extract”[Tiab]) OR (“Asparagus juice”[Tiab]) OR (“North-South Two Bamboo Shoots Recipe”[Tiab]) OR (“Asparagus Chicken Broth Nutritional Health Oral Liquid”[Tiab]) OR (“Asparagus Anticancer Nutritional Oral Liquid”[Tiab]) OR (“asparagus soda”[Tiab])

#3 #1 AND #2
