# Supplementary material for: Asparagusofficinalis potentially supports cancer care: a systematic review of randomized and non-randomized clinical studies
Source: Front Nutr. 2026 Mar 12;13:1621710. doi: 10.3389/fnut.2026.1621710 (PMC13017270; doi:10.3389/fnut.2026.1621710)
Supplement: Supplementary file 2 [file Table_2.DOCX]

**sTable 1. Methodology quality evaluation of cohort study via Newcastle-Ottawa Scale**

**Study ID: Lv QJ 2000**

| **Evaluation item** | | | **Score** |
| --- | --- | --- | --- |
| **Selection** | **1) Representativeness of the exposed cohort** | a) truly representative of the average _______________ (describe) in the community (1) b) somewhat representative of the average ______________ in the community (1) c) selected group of users eg nurses, volunteers (0) d) no description of the derivation of the cohort (0) | c |
|  | **2) Selection of the non-exposed cohort** | a) drawn from the same community as the exposed cohort (1) b) drawn from a different source (0) c) no description of the derivation of the non exposed cohort (0) | a |
|  | **3) Ascertainment of exposure** | a) secure record (eg surgical records) (1) b) structured interview (1) c) written self report (0) d) no description (0) | b |
|  | **4) Demonstration that outcome of interest was not present at start of study** | a) yes (1) b) no (0) | a |
| **Comparability** | **1) Comparability of cohorts on the basis of the design or analysis** | a) study controls for _____________ (select the most important factor) (1)  b) study controls for any additional factor (This criteria could be modified to indicate specific control for a second important factor.) (1) | **ab** |
| **Outcome** | **1) Assessment of outcome** | a) independent blind assessment (1)  b) record linkage (1)  c) self report (0)  d) no description (0) | d |
|  | **2) Was follow-up long enough for outcomes to occur** | a) yes (select an adequate follow up period for outcome of interest) (1)  b) no (0) | b |
|  | **3) Adequacy of follow up of cohorts** | a) complete follow up - all subjects accounted for (1)  b) subjects lost to follow up unlikely to introduce bias - small number lost - > ____ % (select an adequate %) follow up, or description provided of those lost) (1)  c) follow up rate < ____% (select an adequate %) and no description of those lost (0)  d) no statement (0) | d |
| **Total score** | | | 5 |

| **sTable 2. Methodology quality evaluation of non-randomized trials via Non-randomized Studies of Interventions (ROBINS-I)** **tool** | | | | | | |
| --- | --- | --- | --- | --- | --- | --- |
| **Study ID Li XZ 1994** | |  | | | | |
|  | **Signalling questions** | | | **Response options** |  |  |
| **Bias due to confounding** | | | | |  |  |
|  | 1.1 Is there potential for confounding of the effect of intervention in this study?  **If N/PN to 1.1:** the study can be considered to be at low risk of bias due to confounding and no further signalling questions need be considered | | PY | | |  |
|  | **If Y/PY to 1.1**: determine whether there is a need to assess time-varying confounding: | |  | | |  |
|  | 1.2. Was the analysis based on splitting participants’ follow up time according to intervention received?  **If N/PN**, answer questions relating to baseline confounding (1.4 to 1.6)  **If Y/PY**, go to question 1.3. | | NI | | |  |
|  | 1.3. Were intervention discontinuations or switches likely to be related to factors that are prognostic for the outcome?  **If N/PN**, answer questions relating to baseline confounding (1.4 to 1.6)  **If Y/PY**, answer questions relating to both baseline and time-varying confounding (1.7 and 1.8) | | NI | | |  |

|  | **Questions relating to baseline confounding only** | | |
| --- | --- | --- | --- |
|  | 1.4. Did the authors use an appropriate analysis method that controlled for all the important confounding domains? | NI |  |
|  | 1.5. **If Y/PY to 1.4**: Were confounding domains that were controlled for measured validly and reliably by the variables available in this study? | NA |  |
|  | 1.6. Did the authors control for any post-intervention variables that could have been affected by the intervention? | NI |  |
|  | **Questions relating to baseline and time-varying confounding** |  |  |
|  | 1.7. Did the authors use an appropriate analysis method that controlled for all the important confounding domains and for time-varying confounding? | NI |  |
|  | 1.8. **If Y/PY to 1.7**: Were confounding domains that were controlled for measured validly and reliably by the variables available in this study? | NA |  |
|  | **Risk of bias judgement** | Serious |  |

| **Bias in selection of participants into the study** | | | |
| --- | --- | --- | --- |
|  | 2.1. Was selection of participants into the study (or into the analysis) based on participant characteristics observed after the start of intervention?  **If N/PN to 2.1:** go to 2.4 | NI |  |
|  | 2.2. **If Y/PY to 2.1**: Were the post-intervention variables that influenced selection likely to be associated with intervention?  2.3 **If Y/PY to 2.2**: Were the post-intervention variables that influenced selection likely to be influenced by the outcome or a cause of the outcome? | NA  NA |  |
|  | 2.4. Do start of follow-up and start of intervention coincide for most participants? | NI |  |
|  | 2.5. **If Y/PY to 2.2 and 2.3, or N/PN to 2.4**: Were adjustment techniques used that are likely to correct for the presence of selection biases? | NA |  |
|  | **Risk of bias judgement** | NI |  |

| **Bias in classification of interventions** | | | |
| --- | --- | --- | --- |
|  | 3.1 Were intervention groups clearly defined? | PY |  |
|  | 3.2 Was the information used to define intervention groups recorded at the start of the intervention? | NI |  |
|  | 3.3 Could classification of intervention status have been affected by knowledge of the outcome or risk of the outcome? | PY |  |
|  | **Risk of bias judgement** | Moderate |  |

| **Bias due to deviations from intended interventions** | | | |
| --- | --- | --- | --- |
|  | **If your aim for this study is to assess the effect of assignment to intervention, answer questions 4.1 and 4.2** | | |
|  | 4.1. Were there deviations from the intended intervention beyond what would be expected in usual practice? | PN |  |
|  | 4.2. **If Y/PY to 4.1**: Were these deviations from intended intervention unbalanced between groups *and* likely to have affected the outcome? | NA |  |
|  | **If your aim for this study is to assess the effect of starting and adhering to intervention, answer questions 4.3 to 4.6** |  |  |
|  | 4.3. Were important co-interventions balanced across intervention groups? | PN |  |
|  | 4.4. Was the intervention implemented successfully for most participants? | NI |  |
|  | 4.5. Did study participants adhere to the assigned intervention regimen? | NI |  |
|  | 4.6. **If N/PN to 4.3, 4.4 or 4.5**: Was an appropriate analysis used to estimate the effect of starting and adhering to the intervention? | NI |  |
|  | **Risk of bias judgement** | Moderate |  |

| **Bias due to missing data** | | | |
| --- | --- | --- | --- |
|  | 5.1 Were outcome data available for all, or nearly all, participants? | NI |  |
|  | 5.2 Were participants excluded due to missing data on intervention status? | NI |  |
|  | 5.3 Were participants excluded due to missing data on other variables needed for the analysis? | NI |  |
|  | 5.4 **If PN/N to 5.1, or Y/PY to 5.2 or 5.3**: Are the proportion of participants and reasons for missing data similar across interventions? | NA |  |
|  | 5.5 **If PN/N to 5.1, or Y/PY to 5.2 or 5.3**: Is there evidence that results were robust to the presence of missing data? | NA |  |
|  | **Risk of bias judgement** | NI |  |

| **Bias in measurement of outcomes** | | | |
| --- | --- | --- | --- |
|  | 6.1 Could the outcome measure have been influenced by knowledge of the intervention received? | PN |  |
|  | 6.2 Were outcome assessors aware of the intervention received by study participants? | NI |  |
|  | 6.3 Were the methods of outcome assessment comparable across intervention groups? | PY |  |
|  | 6.4 Were any systematic errors in measurement of the outcome related to intervention received? | NI |  |
|  | **Risk of bias judgement** | Moderate |  |

| **Bias in selection of the reported result** | | |
| --- | --- | --- |
|  | Is the reported effect estimate likely to be selected, on the basis of the results, from... |  |
|  | 7.1. ... multiple outcome *measurements* within the outcome domain? | N |
|  | 7.2 ... multiple *analyses* of the intervention-outcome relationship? | N |
|  | 7.3 ... different *subgroups*? | N |
|  | **Risk of bias judgement** | Low |

| **Overall bias** | | |
| --- | --- | --- |
|  | **Risk of bias judgement** | Serious |

| **S2 Table Methodology quality evaluation of non-randomized trials via Non-randomized Studies of Interventions (ROBINS-I)** **tool** | | | | | | |
| --- | --- | --- | --- | --- | --- | --- |
| **ID Wang BC 1996** | |  | | | | |
|  | **Signalling questions** | | | **Response options** |  |  |
| **Bias due to confounding** | | | | |  |  |
|  | 1.1 Is there potential for confounding of the effect of intervention in this study?  **If N/PN to 1.1:** the study can be considered to be at low risk of bias due to confounding and no further signalling questions need be considered | | PN | | |  |
|  | **If Y/PY to 1.1**: determine whether there is a need to assess time-varying confounding: | |  | | |  |
|  | 1.2. Was the analysis based on splitting participants’ follow up time according to intervention received?  **If N/PN**, answer questions relating to baseline confounding (1.4 to 1.6)  **If Y/PY**, go to question 1.3. | | NI | | |  |
|  | 1.3. Were intervention discontinuations or switches likely to be related to factors that are prognostic for the outcome?  **If N/PN**, answer questions relating to baseline confounding (1.4 to 1.6)  **If Y/PY**, answer questions relating to both baseline and time-varying confounding (1.7 and 1.8) | | NI | | |  |

|  | **Questions relating to baseline confounding only** | | |
| --- | --- | --- | --- |
|  | 1.4. Did the authors use an appropriate analysis method that controlled for all the important confounding domains? | NI |  |
|  | 1.5. **If Y/PY to 1.4**: Were confounding domains that were controlled for measured validly and reliably by the variables available in this study? | NA |  |
|  | 1.6. Did the authors control for any post-intervention variables that could have been affected by the intervention? | NI |  |
|  | **Questions relating to baseline and time-varying confounding** |  |  |
|  | 1.7. Did the authors use an appropriate analysis method that controlled for all the important confounding domains and for time-varying confounding? | NI |  |
|  | 1.8. **If Y/PY to 1.7**: Were confounding domains that were controlled for measured validly and reliably by the variables available in this study? | NA |  |
|  | **Risk of bias judgement** | NI |  |

| **Bias in selection of participants into the study** | | | |
| --- | --- | --- | --- |
|  | 2.1. Was selection of participants into the study (or into the analysis) based on participant characteristics observed after the start of intervention?  **If N/PN to 2.1:** go to 2.4 | PN |  |
|  | 2.2. **If Y/PY to 2.1**: Were the post-intervention variables that influenced selection likely to be associated with intervention?  2.3 **If Y/PY to 2.2**: Were the post-intervention variables that influenced selection likely to be influenced by the outcome or a cause of the outcome? | NA  NA |  |
|  | 2.4. Do start of follow-up and start of intervention coincide for most participants? | PY |  |
|  | 2.5. **If Y/PY to 2.2 and 2.3, or N/PN to 2.4**: Were adjustment techniques used that are likely to correct for the presence of selection biases? | NA |  |
|  | **Risk of bias judgement** | Low |  |

| **Bias in classification of interventions** | | | |
| --- | --- | --- | --- |
|  | 3.1 Were intervention groups clearly defined? | Y |  |
|  | 3.2 Was the information used to define intervention groups recorded at the start of the intervention? | PY |  |
|  | 3.3 Could classification of intervention status have been affected by knowledge of the outcome or risk of the outcome? | PY |  |
|  | **Risk of bias judgement** | Moderate |  |

| **Bias due to deviations from intended interventions** | | | |
| --- | --- | --- | --- |
|  | **If your aim for this study is to assess the effect of assignment to intervention, answer questions 4.1 and 4.2** | | |
|  | 4.1. Were there deviations from the intended intervention beyond what would be expected in usual practice? | PN |  |
|  | 4.2. **If Y/PY to 4.1**: Were these deviations from intended intervention unbalanced between groups *and* likely to have affected the outcome? | NA |  |
|  | **If your aim for this study is to assess the effect of starting and adhering to intervention, answer questions 4.3 to 4.6** |  |  |
|  | 4.3. Were important co-interventions balanced across intervention groups? | PY |  |
|  | 4.4. Was the intervention implemented successfully for most participants? | NI |  |
|  | 4.5. Did study participants adhere to the assigned intervention regimen? | NI |  |
|  | 4.6. **If N/PN to 4.3, 4.4 or 4.5**: Was an appropriate analysis used to estimate the effect of starting and adhering to the intervention? | NA |  |
|  | **Risk of bias judgement** | Low |  |

| **Bias due to missing data** | | | |
| --- | --- | --- | --- |
|  | 5.1 Were outcome data available for all, or nearly all, participants? | NI |  |
|  | 5.2 Were participants excluded due to missing data on intervention status? | NI |  |
|  | 5.3 Were participants excluded due to missing data on other variables needed for the analysis? | NI |  |
|  | 5.4 **If PN/N to 5.1, or Y/PY to 5.2 or 5.3**: Are the proportion of participants and reasons for missing data similar across interventions? | NA |  |
|  | 5.5 **If PN/N to 5.1, or Y/PY to 5.2 or 5.3**: Is there evidence that results were robust to the presence of missing data? | NA |  |
|  | **Risk of bias judgement** | NI |  |

| **Bias in measurement of outcomes** | | | |
| --- | --- | --- | --- |
|  | 6.1 Could the outcome measure have been influenced by knowledge of the intervention received? | PN |  |
|  | 6.2 Were outcome assessors aware of the intervention received by study participants? | PY |  |
|  | 6.3 Were the methods of outcome assessment comparable across intervention groups? | PY |  |
|  | 6.4 Were any systematic errors in measurement of the outcome related to intervention received? | NI |  |
|  | **Risk of bias judgement** | Moderate |  |

| **Bias in selection of the reported result** | | |
| --- | --- | --- |
|  | Is the reported effect estimate likely to be selected, on the basis of the results, from... |  |
|  | 7.1. ... multiple outcome *measurements* within the outcome domain? | N |
|  | 7.2 ... multiple *analyses* of the intervention-outcome relationship? | N |
|  | 7.3 ... different *subgroups*? | N |
|  | **Risk of bias judgement** | Low |

| **Overall bias** | | |
| --- | --- | --- |
|  | **Risk of bias judgement** | NI |
