# Supplementary material for: Asparagusofficinalis potentially supports cancer care: a systematic review of randomized and non-randomized clinical studies
Source: Front Nutr. 2026 Mar 12;13:1621710. doi: 10.3389/fnut.2026.1621710 (PMC13017270; doi:10.3389/fnut.2026.1621710)
Supplement: Supplementary file 3 [file Table_3.DOCX]

**sTable 3.** The GRADE evidence profile for Asparagus officinalis for cancer care.

| **Quality assessment** | | | | | | | **No of patients** | | **Effect** | | **Quality** | **Importance** |
| --- | --- | --- | --- | --- | --- | --- | --- | --- | --- | --- | --- | --- |
|  |  |  |  |  |  |  |  |  |  |  |  |  |
| **No of studies** | **Design** | **Risk of bias** | **Inconsistency** | **Indirectness** | **Imprecision** | **Other considerations** | **Asparagus officinalis based intervention** | **Control** | **Relative (95% CI)** | **Absolute** |  |  |
| **One-Year Survival Rate - Asparagus officinalis syrup plus radiotherapy VS radiotherapy** | | | | | | | | | | | | |
| 1 | RCT | serious^1^ | no serious inconsistency | no serious indirectness | serious^2^ | none | 64 | 64 | RR 1.62  (1.14 to 2.28) | 658 per 1,000  (463 to 926) | ⊕⊕OO LOW | IMPORTANT |
| **Survival-Based Effective Rate - Asparagus officinalis granules plus chemotherapy VS chemotherapy** | | | | | | | | | | | | |
| 2 | RCT | serious^1^ | no serious inconsistency | no serious indirectness | serious^2^ | none | 168 | 84 | RR 1.55  (1.24 to 1.92) | 812 per 1,000  (650 to 1,000) | ⊕⊕OO LOW | IMPORTANT |
| **Objective Response Rate - Asparagus officinalis based intervention plus chemotherapy VS chemotherapy** | | | | | | | | | | | | |
| 4 | RCT | serious^1^ | no serious inconsistency | no serious indirectness | serious^2^ | none | 254 | 168 | RR 1.88 (1.43 to 2.48) | 515 per 1,000 (392 to 679) | ⊕⊕OO LOW | IMPORTANT |
| **Disease Control Rate - Asparagus officinalis syrup plus chemotherapy VS chemotherapy** | | | | | | | | | | | | |
| 1 | RCT | serious^1^ | no serious inconsistency | no serious indirectness | very serious^2,3^ | none | 26 | 24 | RR 1.15 (0.95 to 1.40) | 958 per 1,000 (792 to 1,000) | ⊕OOO VERY LOW | IMPORTANT |
| **Karnofsky Score - Asparagus officinalis syrup plus chemotherapy VS chemotherapy** | | | | | | | | | | | | |
| 1 | RCT | serious^1^ | no serious inconsistency | no serious indirectness | serious^2^ | none | 28 | 29 | MD 7.95 higher (3.83 higher to 12.07 higher) | —— | ⊕⊕OO LOW | IMPORTANT |
| **Quality-of-Life-Based Effective Rate - Asparagus officinalis granules plus chemotherapy VS chemotherapy** | | | | | | | | | | | | |
| 3 | RCT | serious^4^ | no serious inconsistency | no serious indirectness | serious^2^ | none | 228 | 144 | RR 1.76 (1.47 to 2.11) | 843 per 1,000 (704 to 1,000) | ⊕⊕OO LOW | IMPORTANT |

^1^ High risk of bias or some concern.

^2^ The total sample size for continuous data is less than 400 or the total number of events for Dichotomous data is less than 300.

^3^ Wide confidence intervals around the estimate of the effect.

**sTable 4. Meta-analysis results of immune function**

| **Comparisons** | **Time points of the outcome measured (days)** | **Effect size** | **95% confidence interval** | **№ of participants (studies)** |
| --- | --- | --- | --- | --- |
| **Outcome 1 CD3** | | | | |
| Asparagus officinalis granules + radiotherapy VS radiotherapy | 15 | MD 1.77 | -1.45 to 4.99 | 40 (1 study (44)) |
| Asparagus officinalis granules + radiotherapy VS radiotherapy | 30 | MD 7.99 | **4.11 to 11.87＊** | 40 (1 study (44)) |
| Asparagus officinalis granules + radiotherapy VS radiotherapy | 45 | MD 12.9 | **9.82 to 15.98＊** | 40 (1 study (44)) |
| Asparagus officinalis granules + chemotherapy VS chemotherapy | 60 | MD 17.26 | **15.42 to 19.1＊** | 252 (2 studies (41, 46)) |
| **Outcome 2 CD4** | | | | |
| Asparagus officinalis granules + radiotherapy VS radiotherapy | 15 | MD 1.47 | -1.59 to 4.53 | 40 (1 study (44)) |
| Asparagus officinalis granules + radiotherapy VS radiotherapy | 30 | MD 7.41 | **3.73 to 11.09＊** | 40 (1 study (44)) |
| Asparagus officinalis granules + radiotherapy VS radiotherapy | 45 | MD 9.77 | **6.81 to 12.73＊** | 40 (1 study (44)) |
| Asparagus officinalis oral liquid + chemotherapy VS chemotherapy | 45 | MD 6.09 | **4.67 to 7.51＊** | 57 (1 study (43)) |
| Asparagus officinalis granules + chemotherapy VS chemotherapy | 60 | MD 8.6 | **6.42 to 10.78＊** | 252 (2 studies (41, 46)) |
| **Outcome 3 CD8** | | | | |
| Asparagus officinalis granules + radiotherapy VS radiotherapy | 15 | MD 0.19 | -3.31 to 3.69 | 40 (1 study (44)) |
| Asparagus officinalis granules + radiotherapy VS radiotherapy | 30 | MD 0.01 | -3.17 to 3.15 | 40 (1 study (44)) |
| Asparagus officinalis granules + radiotherapy VS radiotherapy | 45 | MD 0.68 | -4.12 to 2.76 | 40 (1 study (44)) |
| Asparagus officinalis oral liquid + chemotherapy VS chemotherapy | 45 | MD 5.12 | **-6.43 to -3.81＊** | 57 (1 study (43)) |
| Asparagus officinalis granules + chemotherapy VS chemotherapy | 60 | MD 2 | **0.28 to 3.72＊** | 252 (2 studies (41, 46)) |
| **Outcome 4 CD4/CD8** | | | | |
| Asparagus officinalis granules + radiotherapy VS radiotherapy | 15 | MD 0.06 | -0.23 to 0.35 | 40 (1 study (44)) |
| Asparagus officinalis granules + radiotherapy VS radiotherapy | 30 | MD 0.38 | **0.03 to 0.73＊** | 40 (1 study (44)) |
| Asparagus officinalis granules + radiotherapy VS radiotherapy | 45 | MD 0.59 | **0.19 to 0.99＊** | 40 (1 study (44)) |
| Asparagus officinalis oral liquid + chemotherapy VS chemotherapy | 45 | MD 0.39 | **0.27 to 0.51＊** | 57 (1 study (43)) |
| Asparagus officinalis granules + chemotherapy VS chemotherapy | 60 | MD 0.2 | **0.08 to 0.33＊** | 252 (2 studies (41, 46)) |
| **Outcome 5 NK cell activity** | | | | |
| Asparagus officinalis granules + chemotherapy VS chemotherapy | 20 | MD 4 | **0.92 to 7.08＊** | 20 (1 study (44)) |
| Asparagus officinalis granules + chemotherapy VS chemotherapy | 47 | MD 10.5 | **7.32 to 13.68＊** | 20 (1 study (44)) |
| Asparagus officinalis granules + chemotherapy VS chemotherapy | 60 | MD 11.73 | **9.86 to 13.6＊** | 372 (3 studies (39, 41, 46)) |
| **Outcome 6 LAK cell activity** | | | | |
| Asparagus officinalis granules + chemotherapy VS chemotherapy | 60 | MD 11.27 | **9.57 to 12.97＊** | 252 (2 studies (41, 46)) |
| **Outcome 7 Platelet reduction degree** | | | | |
| **Outcome 7.1 Platelet reduction degree, Grade 0: ≥100 × 10^9/L** | | | | |
| Asparagus officinalis syrup + chemotherapy VS chemotherapy | NR | RR 1.38 | 0.78 to 2.46 | 50 (1 study (42)) |
| **Outcome 7.2 Platelet reduction degree, Grade 1: (75-99) × 10^9/L** | | | | |
| Asparagus officinalis syrup + chemotherapy VS chemotherapy | NR | RR 0.82 | 0.38 to 1.78 | 50 (1 study (42)) |
| **Outcome 7.3 Platelet reduction degree, Grade 2: (50-74) × 10^9/L** | | | | |
| Asparagus officinalis syrup + chemotherapy VS chemotherapy | NR | RR 1.38 | 0.25 to 7.59 | 50 (1 study (42)) |
| **Outcome 7.4 Platelet reduction degree, Grade 3: (25-49) × 10^9/L** | | | | |
| Asparagus officinalis syrup + chemotherapy VS chemotherapy | NR | RR 0.13 | 0.01 to 2.44 | 50 (1 study (42)) |
| **Outcome 7.5 Platelet reduction degree, Grade 4: <25 × 10^9/L** | | | | |
| Asparagus officinalis syrup + chemotherapy VS chemotherapy | NR | not estimable (0 events) |  | 50 (1 study (42)) |
| **Outcome 8 Red blood cell reduction** | | | | |
| **Outcome 8.1 Red blood cell reduction: total number of cases** | | | | |
| Asparagus officinalis granules + chemotherapy VS chemotherapy | NR | RR 0.57 | 0.25 to 1.28 | 45 (1 study (44)) |
| **Outcome 8.2 Red blood cell reduction: <2.3 × 10^12/L** | | | | |
| Asparagus officinalis granules + chemotherapy VS chemotherapy | NR | RR 0.21 | 0.01 to 4.12 | 45 (1 study (44)) |
| **Outcome 8.3 Red blood cell reduction: (2.9-2.4) × 10^12/L** | | | | |
| Asparagus officinalis granules + chemotherapy VS chemotherapy | NR | RR 0.63 | 0.17 to 2.32 | 45 (1 study (44)) |
| **Outcome 8.4 Red blood cell reduction: (3.5-3.0) × 10^12/L** | | | | |
| Asparagus officinalis granules + chemotherapy VS chemotherapy | NR | RR 0.78 | 0.20 to 3.11 | 45 (1 study (44)) |
| **Outcome 9 Leukocyte reduction degree** | | | | |
| **Outcome 9.1 Leukocyte reduction degree, Grade 0: ≥4.0 × 10^9/L** | | | | |
| Asparagus officinalis syrup + chemotherapy VS chemotherapy | NR | RR 2.15 | 0.99 to 4.69 | 50 (1 study (42)) |
| **Outcome 9.2 Leukocyte reduction degree, Grade 1: (3.0-3.9) × 10^9/L** | | | | |
| Asparagus officinalis syrup + chemotherapy VS chemotherapy | NR | RR 1.23 | 0.50 to 3.03 | 50 (1 study (42)) |
| Asparagus officinalis granules + chemotherapy VS chemotherapy | NR | RR 1.25 | 0.45 to 3.52 | 45 (1 study (44)) |
| Sub-total effect size | NR | RR 1.24 | 0.63 to 2.45 | 95 (2 studies (42, 44)) |
| **Outcome 9.3 Leukocyte reduction degree, Grade 2: (2.0-2.9) × 10^9/L** | | | | |
| Asparagus officinalis syrup + chemotherapy VS chemotherapy | NR | RR 0.55 | 0.15 to 2.07 | 50 (1 study (42)) |
| Asparagus officinalis granules + chemotherapy VS chemotherapy | NR | RR 1.29 | 0.83 to 2.00 | 45 (1 study (44)) |
| Sub-total effect size | NR | RR 1.04 | 0.48 to 2.24 | 95 (2 studies (42, 44)) |
| **Outcome 9.4 Leukocyte reduction degree, Grade 3: (1.0 - 1.9) × 10^9/L** | | | | |
| Asparagus officinalis syrup + chemotherapy VS chemotherapy | NR | RR 0.31 | 0.03 to 2.76 | 50 (1 study (42)) |
| Asparagus officinalis granules + chemotherapy VS chemotherapy | NR | RR 0.09 | 0.01 to 1.62 | 45 (1 study (44)) |
| Sub-total effect size | NR | RR 0.20 | 0.03 to 1.12 | 95 (2 studies (42, 44)) |
| **Outcome 9.5 Leukocyte reduction degree, Grade 4: <1.0 × 10^9/L** | | | | |
| Asparagus officinalis syrup + chemotherapy VS chemotherapy | NR | not estimable (0 event) |  | 50 (1 study (42)) |
| **Outcome 10** **Blood Non-specific α- Esterase Staining T - Lymphocyte Count (ANAE)** | | | | |
| Asparagus officinalis syrup + chemotherapy VS chemotherapy | NR | MD **7.09** | **1.98 to 12.2＊** | 35 (1 study (42)) |
| **Outcome 11 OKT1 Value of T - Cell Subsets** | | | | |
| Asparagus officinalis syrup + chemotherapy VS chemotherapy | NR | MD 4.05 | **0.39 to 7.71＊** | 35 (1 study (42)) |

**＊The effects show significant differences.**
